# Sleep is enhanced in aged male mice that overexpress calcium/calmodulin-dependent protein kinase IV

**DOI:** 10.3389/fnins.2025.1596602

**Published:** 2025-06-03

**Authors:** Sierra P. Feeney, Erin Threlfall, James M. Bilboa, Christopher C. Angelakos, Mathieu E. Wimmer, Satoshi Kida, Ted Abel, Jennifer C. Tudor

**Affiliations:** ^1^Department of Biology, Saint Joseph’s University, Philadelphia, PA, United States; ^2^Department of Biology, University of Pennsylvania, Philadelphia, PA, United States; ^3^Department of Psychology and Neuroscience, Temple University, Philadelphia, PA, United States; ^4^Department of Bioscience, Graduate School of Agriculture and Life Sciences, University of Tokyo, Tokyo, Japan; ^5^Department of Neuroscience and Pharmacology, Carver College of Medicine, University of Iowa, Iowa City, IA, United States; ^6^Iowa Neuroscience Institute, University of Iowa, Iowa City, IA, United States

**Keywords:** aging, circadian rhythms and sleep, cyclic-AMP response element binding protein, electroencephalography, memory

## Abstract

The dysregulation of sleep–wake patterns that occurs during aging is well documented and coincides with changes in intracellular signaling pathways that regulate sleep, such as the calcium/calmodulin-dependent protein kinase (CaMKII)/cyclic-AMP response element-binding protein (CREB) pathway. However, much less is known about the relationship between other CREB-activating members of the CaMK family, such as calcium/calmodulin-dependent protein kinase IV (CaMKIV), and the regulation of sleep. Using 2- to 4-month-old (young adult) and 22- to 24-month-old (aged) male and female CaMKIV-overexpressing (CaMKIV-OE) mice, we observed that overexpression of CaMKIV in the forebrain decreased wakefulness and increased the amount of non-rapid eye movement (NREM) and rapid eye movement (REM) sleep in aged male mice, but not young adult male mice, in comparison to age- and sex-matched controls. Conversely, female mice overexpressing CaMKIV displayed no significant differences in the percentage of time spent in each vigilance state compared to their wild-type counterparts, regardless of age. While CaMKIV overexpression also led to more sleep–wake fragmentation in young adult and aged male mice, aged female mice displayed more consolidated NREM sleep. Overall, our results suggest that CaMKIV overexpression enhances sleep in aged male mice, and differentially affects sleep–wake architecture based on sex and age, providing insights into the potential mechanism by which CaMKIV overexpression enhances memory.

## 1 Introduction

Over the past decade, the percentage of individuals aged 65 and older has increased from 13.7 to 17% in the U.S. alone and is expected to reach 22% by 2040 (US Department of Health and Human Services A Profile of Older Americans: 2023, 2024; US Department of Health and Human Services A Profile of Older Americans: 2012, n.d.). This demographic shift has created an increased need to understand the distinct physiological changes that occur during aging, such as disruptions in sleep–wake architecture, and their implications for cognitive function. Specifically, healthy aging is commonly characterized by sleep–wake fragmentation, including an increased difficulty initiating and maintaining sleep, as well as an inability to maintain wakefulness during the active phase (Mander et al., 2017; McKillop and Vyazovskiy, 2020). A reduction in non-rapid eye movement (NREM) sleep, primarily including decreased time spent in slow wave sleep, as well as reduced NREM spectral power and sleep spindles, has also been noted during aging (Mander et al., 2017; McKillop and Vyazovskiy, 2020). These age-related changes in sleep–wake architecture coincide with changes in intracellular signaling pathways important for memory and cognition, such as the cyclic-AMP response element-binding protein (CREB) pathway (Yu et al., 2017).

CREB-mediated transcription is normally activated by the cAMP-PKA and CaMK pathways, which are dependent on calcium signaling (Mayr and Montminy, 2001; Lonze and Ginty, 2002). However, during aging, CREB levels and activity decrease in the brain, concurrent with disruptions in calcium buffering and homeostasis (Toescu and Vreugdenhil, 2010; Yu et al., 2017). These age-induced changes in CREB activity are mediated in part by decreased expression of CaMKIV, a serine/threonine kinase that phosphorylates CREB at Serine 133 in response to activity-induced increases in intracellular calcium levels (Matthews et al., 1994; Lu et al., 2004; Fukushima et al., 2008; Loerch et al., 2008). Phosphorylation at Serine 133 then promotes CREB binding to its transcriptional co-activator CREB-binding protein (CBP), ultimately leading to the activation of cAMP response element (CRE) mediated transcription through recruitment of CBP to the promoter of CREB target genes (Chrivia et al., 1993; Mayr and Montminy, 2001).

CREB has been implicated in the maintenance of sleep–wake architecture, specifically by promoting and stabilizing wakefulness (Hendricks et al., 2001; Graves et al., 2003; Wimmer et al., 2021). For example, CREB phosphorylation has been shown to increase in the cerebral cortex during wake and decrease during sleep (Cirelli and Tononi, 2000), and mice with forebrain deletion of CREB show increased NREM sleep and decreased wake (Wimmer et al., 2021). Although the role of CaMKIV in sleep–wake regulation remains largely understudied, particularly in the context of aging, CaMKIV overexpression in the forebrain of mice has been shown to enhance cortical slow delta oscillations during post-learning NREM sleep (Steenland et al., 2010). Further, CaMKIV is one of several rat cortical and cerebellar transcripts that is upregulated during periods of sleep independent of time of day (Cirelli et al., 2004), and sleep deprivation has been shown to downregulate basal levels of CaMKIV in the CA1 and dentate gyrus regions of the hippocampus in mice (Zagaar et al., 2013, 2016). Given the connection between CaMKIV and the regulation of sleep, we sought to determine whether CaMKIV overexpression in the forebrain of young adult and aged mice could alter sleep–wake architecture.

## 2 Materials and methods

### 2.1 Animal subjects

Young (2–4 months) and aged (22–24 months) CaMKIV-overexpressing (CaMKIV-OE) mice (*n* = 5 or 7 for each sex, age, and genotype combination) were obtained and housed under standard conditions, with food and water provided *ad libitum*. CaMKIV-OE mice had a C57BL/6N background, with forebrain expression of the CaMKIV transgene driven by a CaMKIIα promoter (Fukushima et al., 2008). Mice were maintained under a 12 h light–dark cycle, with lights on (ZT 0) at 7 AM. All experiments were conducted according to the National Institutes of Health *Guide for the Care and Use of Laboratory Animals* and were approved by the University of Pennsylvania’s Institutional Animal Care and Use Committee.

### 2.2 Electrode implantation surgery

To measure sleep–wake activity, mice underwent surgery under isoflurane anesthesia for the implantation of electroencephalographic (EEG) and electromyographic (EMG) electrodes (Wimmer et al., 2013). Following surgery, mice were given a minimum of 2 weeks to recover. In the second week of recovery, the mice began acclimation with the cables and recording chambers that would be used during testing (Wimmer et al., 2013).

### 2.3 EEG/EMG recordings

EEG signals were filtered at a frequency of 0.3–60.0 Hz (0.5 amplitude, 6 dB/octave), while EMG signals were filtered at a frequency of 1–100 Hz with 12A5 amplifiers (Astro-Med, West Warwick, RI) and sampled at 256 Hz with 12-bit resolution (Graves et al., 2003). Both EEG and EMG signals were recorded over a 24 h period starting at light onset (ZT 0). During this period, the mice were not disturbed. The overall percentage of time spent in each vigilance state was computed for this entire 24 h period, as well as for the 12 h spent in each part of the light–dark cycle, and for each individual hour over the 24 h period. SleepSign software (Kissei Comtec, INC, Japan) was used to visually score the recordings. A trained experimenter blind to the genotypes of the mice manually scored the data using 4-s epochs for wake, NREM sleep, or rapid eye movement (REM) sleep. Sleep–wake architecture was then analyzed through conventional means by recording the total number of bouts in each state and calculating the mean bout duration (in seconds) for each sleep–wake state transition during the 12 h light and 12 h dark periods. Recordings with excessive movement artifacts were excluded from data analysis.

### 2.4 Statistical analysis

Mann–Whitney *U* tests were used to compare the mean percentage of time spent in each vigilance state (wake, NREM sleep, and REM sleep) between CaMKIV-OE mice and wildtype mice for the 24 h cycle, 12 h light period, and 12 h dark period. Vigilance state 24 h distributions calculated per hour were analyzed using two-way repeated measure analyses of variance (ANOVAs) to fit a full model for genotype effects, time cycle effects, and interaction effects. Multiple comparisons were corrected using the Šidák method to compare independent comparisons at each hour across the 24 h distribution. Comparisons for the mean duration of bouts and the number of bouts between the 12 h light and 12 h dark periods were also analyzed using Mann–Whitney *U* tests. Data are reported as means and standard error of the mean (SEM) with the threshold of significance set at 0.05.

## 3 Results

### 3.1 CaMKIV overexpression does not significantly alter the percentage of time spent in each vigilance state in young adult male mice

**Figure 1:**
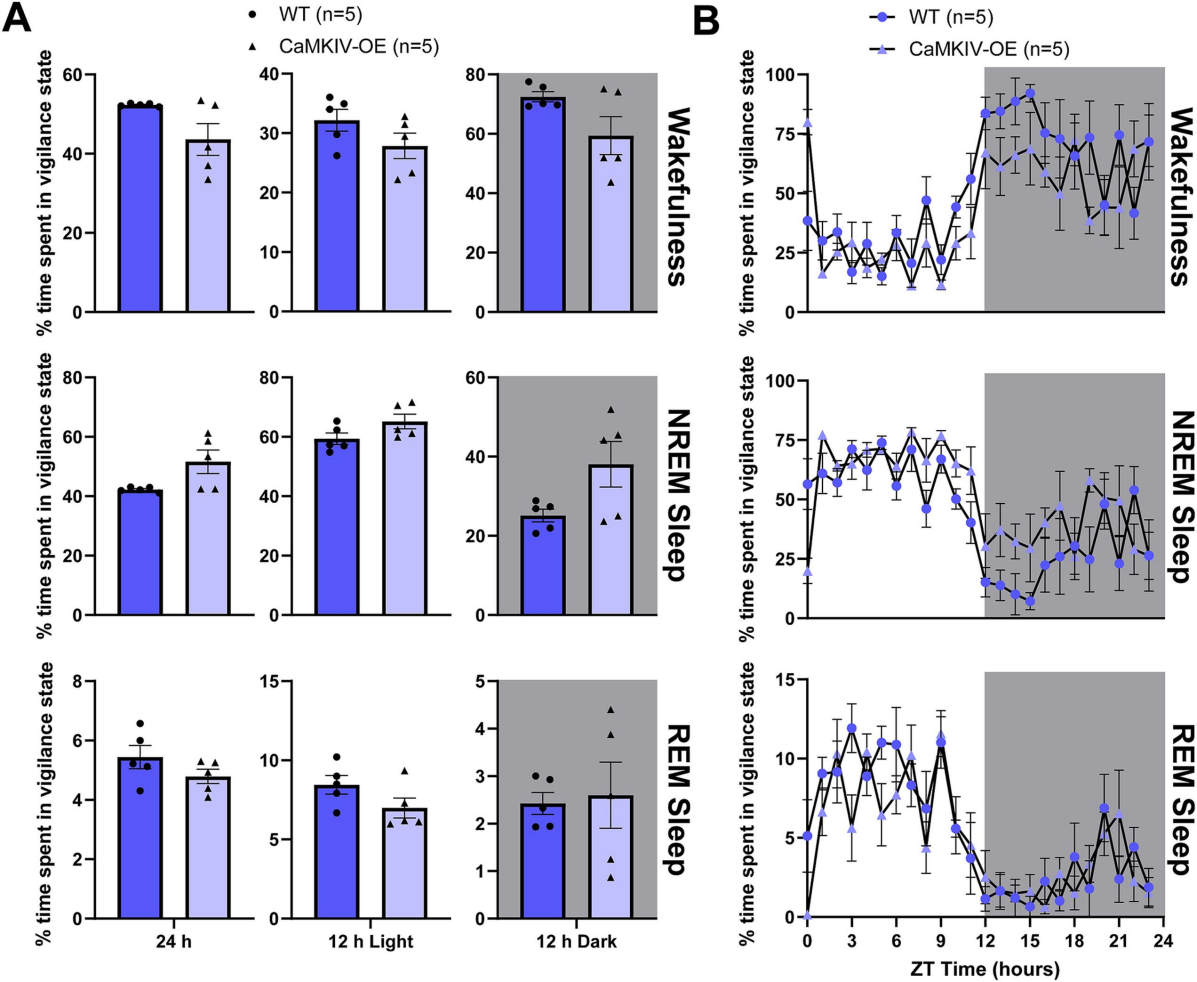
Young adult CaMKIV-OE male mice had no significant differences in the percentage of time spent in each vigilance state for the 24 h cycle, 12 h light period, and 12 h dark period. **(A)** Percentage of time spent in each vigilance state (wake, NREM sleep, and REM sleep) for the total 24 h recording, the 12 h light period, and the 12 h dark period for young adult WT and CaMKIV-OE male mice. No significant genotype effects were found for 24 h wake (*p* = 0.309), 12 h light wake (*p* = 0.151), 12 h dark wake (*p* = 0.222), 24 h NREM sleep (*p* = 0.095), 12 h light NREM sleep (*p* = 0.151), 12 h dark NREM sleep (*p* = 0.222), 24 h REM sleep (*p* = 0.421), 12 h light REM sleep (*p* = 0.151), and 12 h dark REM sleep (*p* > 0.999). Data are means ± SEM of 5 mice in each condition. Significance is based on Mann–Whitney U test comparisons between genotypes. **(B)** Hourly distribution of the percentage of time spent in each vigilance state (wake, NREM sleep, and REM sleep) across the total 24 h recording. For wakefulness, significant time cycle and interaction effects were found (respectively, *F*_23,184_ = 9.653, *p* < 0.0001; *F*_23,184_ = 1.646, *p* = 0.0381), but no significant genotype effect was observed (*F*_1,8_ = 4.731, *p* = 0.0613). For NREM sleep, significant time cycle, genotype, and interaction effects were found (respectively, *F*_23,184_ = 8.937, *p* < 0.0001; *F*_1,8_ = 5.569, *p* = 0.0144; *F*_23,184_ = 1.709, *p* = 0.0281). For REM sleep, a significant time cycle effect was found (*F*_23,184_ = 9.566, *p* < 0.0001), but no significant genotype or interaction effects were observed (respectively, *F*_1,8_ = 2.017, *p* = 0.1933; *F*_23,184_ = 1.332, *p* = 0.1519). Data are means ± SEM of 5 mice in each condition for each hour across the 24 h distribution. Significance is based on 2-way ANOVAs using multiple comparisons. Gray backgrounds indicate the 12 h dark period.

We first assessed how CaMKIV overexpression affects sleep–wake architecture by quantifying the total amount of time young adult male mice spent in each vigilance state. During the 24 h recording period, 12 h light period, and 12 h dark period, young adult CaMKIV-OE male mice displayed no significant differences in the amount of time spent in each of the three vigilance states compared to their young wild-type counterparts (Figure 1A). To explore potential time-of-day effects masked in the broader 12 h intervals, additional analysis was performed in 6 h bins, revealing a significant decrease in wakefulness and increase in NREM sleep in young adult CaMKIV-OE mice during the second half of the light period (Supplementary Figure 1). The 24 h distribution of sleep–wake states was also analyzed by considering each individual hourly value of the percentage of time spent in each vigilance state (Figure 1B). Overall, there were no significant differences between genotypes for all three vigilance states (Figure 1B).

### 3.2 CaMKIV overexpression does not significantly alter the percentage of time spent in each vigilance state in young adult female mice

**Figure 2:**
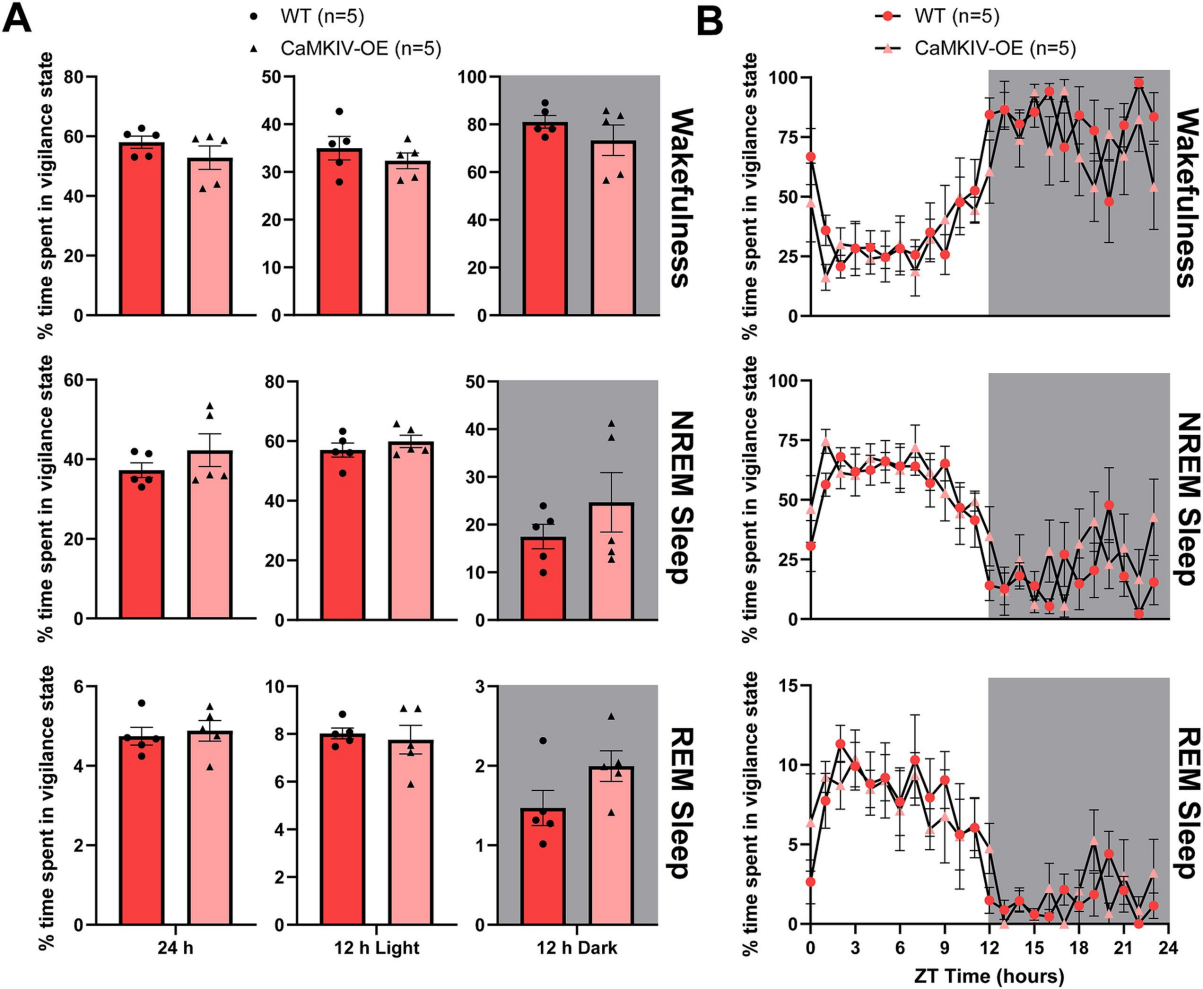
Young adult CaMKIV-OE female mice had no significant differences in the percentage of time spent in each vigilance state for the 24 h cycle, 12 h light period, and 12 h dark period. **(A)** Percentage of time spent in each vigilance state (wake, NREM sleep, and REM sleep) for the total 24 h recording, the 12 h light period, and the 12 h dark period for young adult WT and CaMKIV-OE female mice. No significant genotype effects were observed for 24 h wake (*p* = 0.222), 12 h light wake (*p* = 0.690), 12 h dark wake (*p* = 0.690), 24 h NREM sleep (*p* = 0.421), 12 h light NREM sleep (*p* = 0.421), 12 h dark NREM sleep (*p* = 0.690), 24 h REM sleep (*p* = 0.548), 12 h light REM sleep (*p* = 0.841), and 12 h dark REM sleep (*p* = 0.151). Data are means ± SEM of 5 mice in each condition. Significance is based on Mann–Whitney U test comparisons between genotypes. **(B)** Hourly distribution of the percentage of time spent in each vigilance state (wake, NREM sleep, and REM sleep) across the total 24 h recording. For wakefulness, significant time cycle effects were found (*F*_23,184_ = 10.63, *p* < 0.0001), but no significant genotype or interaction effects were observed (respectively, *F*_1,8_ = 1.367, *p* = 0.276; *F*_23,184_ = 1.041, *p* = 0.417). For NREM sleep, significant time cycle effects were found (*F*_23,184_ = 10.30, *p* < 0.0001), but no significant genotype or interaction effects were observed (respectively, *F*_1,8_ = 1.242, *p* = 0.297; *F*_23,184_ = 1.080, *p* = 0.371). For REM sleep, significant time cycle effects were found (*F*_23,184_ = 8.622, *p* < 0.0001), but no significant genotype or interaction effects were observed (respectively, *F*_1,8_ = 0.1537, *p* = 0.705; *F*_23,184_ = 0.6622, *p* = 0.878). Data are means ± SEM of 5 mice in each condition for each hour across the 24 h distribution. Significance is based on 2-way ANOVAs using multiple comparisons. Gray backgrounds indicate the 12 h dark period.

Knowing how CaMKIV overexpression affects sleep in young adult males, we wanted to examine the same question in their female counterparts. During the full 24 h recording period, 12 h light period, and 12 h dark period, young adult CaMKIV-OE female mice displayed no significant differences in the amount of time spent in each of the three vigilance states compared to young adult wild-type female mice (Figure 2A). To explore potential time-of-day effects masked in the broader 12 h intervals, additional analysis was performed in 6 h bins; however, no significant differences in the amount of time spent in each of the three vigilance states were found (data not shown). Furthermore, when examining the 24 h distribution of sleep–wake states for young adult CaMKIV-OE female mice, no significant differences for each individual hour between genotypes for all three vigilance states were observed (Figure 2B).

### 3.3 CaMKIV overexpression decreases wakefulness and increases the percentage of time spent in NREM and REM sleep in aged male mice

**Figure 3:**
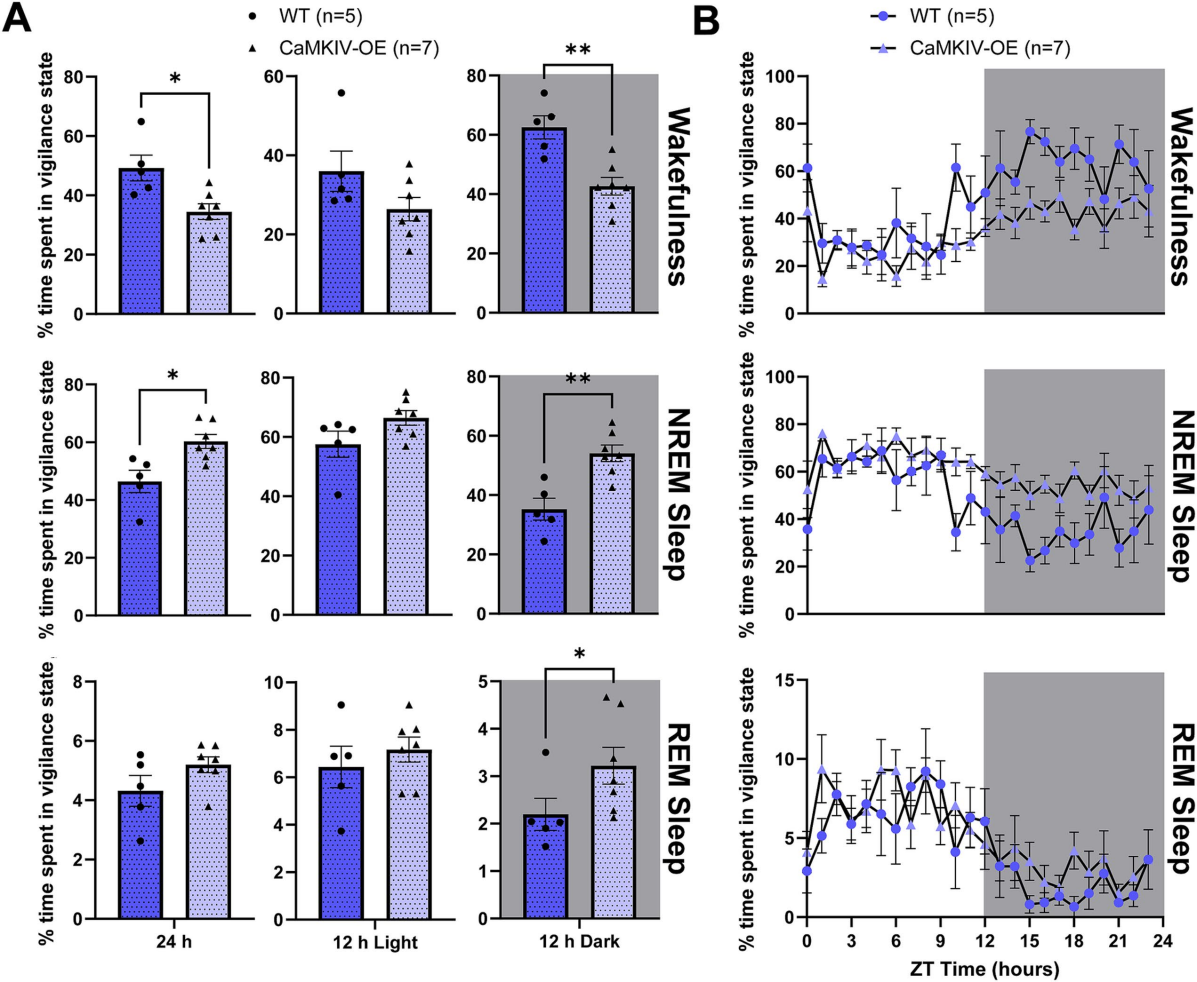
Aged CaMKIV-OE male mice had decreased wakefulness in the 24 h cycle and the 12 h dark period, increased NREM sleep in the 24 h cycle and the 12 h dark period, as well as increased REM sleep during the 12 h dark period. **(A)** Percentage of time spent in each vigilance state (wake, NREM sleep, and REM sleep) for the total 24 h recording, the 12 h light period, and the 12 h dark period for aged WT and CaMKIV-OE male mice. Significant genotype effects were found for 24 h wake (*p = 0.0101), 12 h dark wake (**p = 0.00505), 24 h NREM sleep (*p = 0.0101), 12 h dark NREM sleep (**p = 0.00505), and 12 h dark REM sleep (*p = 0.0480). No significant genotype effects were observed for 12 h light wake (*p* = 0.202), 12 h light NREM sleep (*p* = 0.202), 24 h REM sleep (*p* = 0.202), and 12 h light REM sleep (*p* = 0.407). Data are means ± SEM of 5 or 7 mice in each condition. Asterisks indicate significant comparisons between genotypes (**p < 0.01; *p < 0.05; Mann–Whitney *U* test). **(B)** Hourly distribution of the percentage of time spent in each vigilance state (wake, NREM sleep, and REM sleep) across the total 24 h recording. For wakefulness, significant time cycle and genotype effects were found (respectively, *F*_23,230_ = 5.726, *p* < 0.0001; *F*_1,10_ = 9.452, *p* = 0.0118), but no significant interaction effect was observed (*F*_23,230_ = 0.9231, *p* = 0.5676). For NREM sleep, significant time cycle and genotype effects were found (respectively, *F*_23,230_ = 5.048, *p* < 0.001; *F*_1,10_ = 10.32, *p* = 0.0093), but no significant interaction effect was observed (*F*_23,230_ = 0.9814, *p* = 0.490). For REM sleep, a significant time cycle effect was found (*F*_23,230_ = 5.442, *p* < 0.0001), but no significant genotype or interaction effects were observed (respectively, *F*_1,10_ = 2.659, *p* = 0.1340; *F*_23,230_ = 0.7392, *p* = 0.802). Data are means ± SEM of 5 or 7 mice in each condition for each hour across the 24 h distribution. Significance is based on 2-way ANOVAs using multiple comparisons. Gray backgrounds indicate the 12 h dark period.

We next investigated how sleep–wake architecture is affected in aged mice overexpressing CaMKIV. During the full 24 h recording period, aged CaMKIV-OE male mice spent more time asleep, particularly in NREM sleep (Figure 3A). This decreased time spent in wakefulness and increased time spent in NREM sleep was observed primarily during the 12 h dark period (Figure 3A). Specific to the 12 h dark period, aged CaMKIV-OE male mice also spent more time in REM sleep (Figure 3A). To explore potential time-of-day effects beyond these broader 12 h intervals, additional analysis was performed in 6 h bins, revealing a significant decrease in wakefulness and increase in NREM sleep in aged CaMKIV-OE mice during both the first half of the dark period and the second half of the dark period (Supplementary Figure 2). However, when analyzing each individual hourly value over the 24 h distribution of sleep–wake states for aged CaMKIV-OE male mice, there were no significant differences for each individual hour between genotypes for all three vigilance states (Figure 3B). Taken together, this data likely indicates that although CaMKIV overexpression enhances sleep by increasing the total amount of time spent in NREM sleep, the effect of CaMKIV overexpression has to accumulate over many hours to produce the changes seen over the total 24 h period. When considered alongside the results obtained for their young adult male counterparts, this data also indicates that the effects of CaMKIV overexpression are differential based on age.

### 3.4 CaMKIV overexpression does not significantly alter the percentage of time spent in each vigilance state in aged female mice

**Figure 4:**
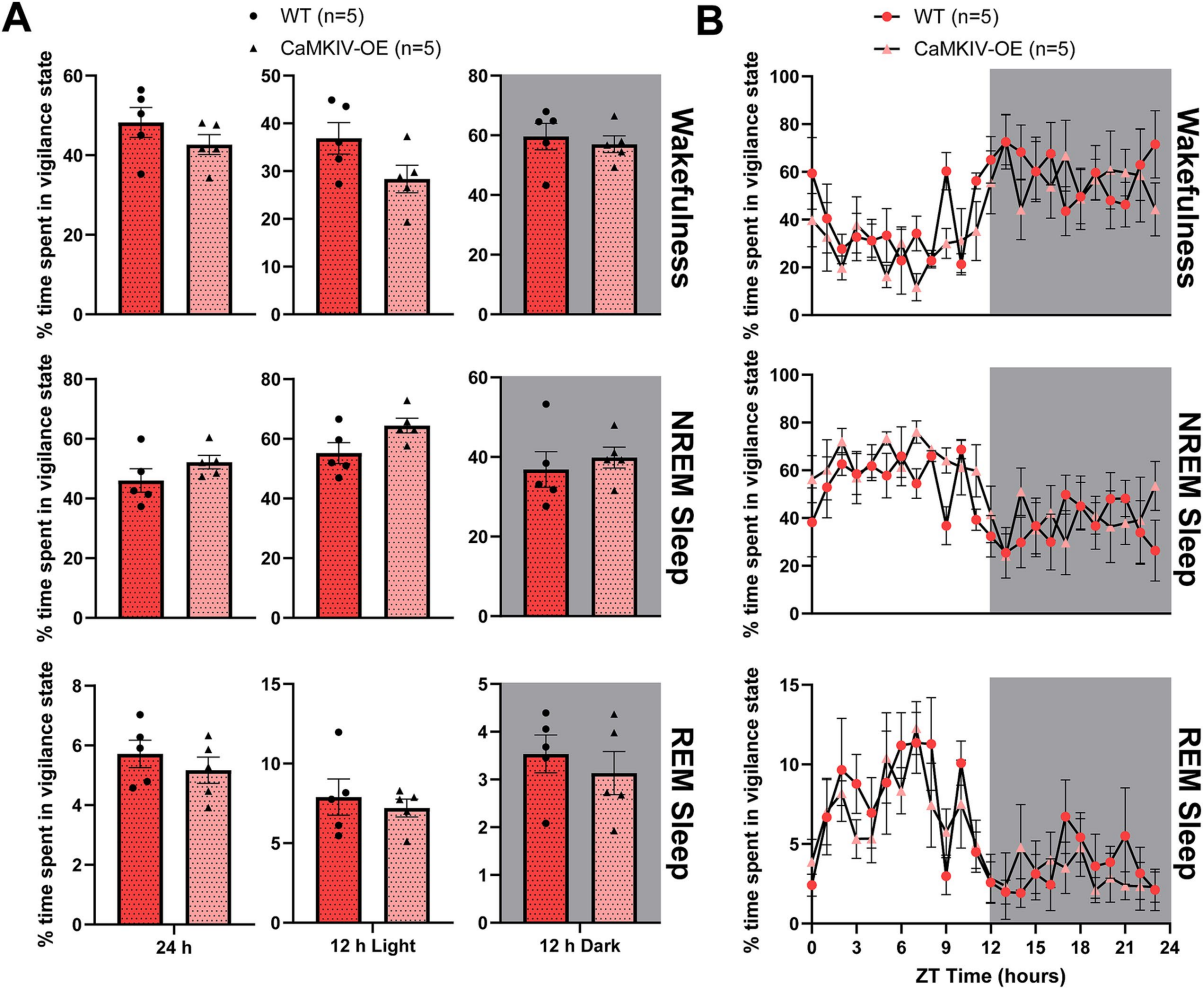
Aged CaMKIV-OE female mice had no significant differences in the percentage of time spent in each vigilance state for the 24 h cycle, 12 h light period, and 12 h dark period. **(A)** Percentage of time spent in each vigilance state (wake, NREM sleep, and REM sleep) for the total 24 h recording, the 12 h light period, and the 12 h dark period for aged WT and CaMKIV-OE female mice. No significant genotype effects were observed for 24 h wake (*p* = 0.222), 12 h light wake (*p* = 0.151), 12 h dark wake (*p* = 0.548), 24 h NREM sleep (*p* = 0.222), 12 h light NREM sleep (*p* = 0.151), 12 h dark NREM sleep (*p* = 0.421), 24 h REM sleep (*p* = 0.421), 12 h light REM sleep (*p* = 0.841), and 12 h dark REM sleep (*p* = 0.548). Data are means ± SEM of 5 or 8 mice in each condition. Significance is based on Mann–Whitney U test comparisons between genotypes. **(B)** Hourly distribution of the percentage of time spent in each vigilance state (wake, NREM sleep, and REM sleep) across the total 24 h recording. For wakefulness, a significant time cycle effect was found (*F*_23,184_ = 3.944, *p* < 0.0001), but no significant genotype or interaction effects were observed (respectively, *F*_1,8_ = 1.516, *p* = 0.253; *F*_23,184_ = 0.8454, *p* = 0.671). For NREM sleep, a significant time cycle effect was found (*F*_23,184_ = 3.472, *p* < 0.0001), but no significant genotype or interaction effects were observed (respectively, *F*_1,8_ = 1.797, *p* = 0.217; *F*_23,184_ = 0.8674, *p* = 0.642). For REM sleep, a significant time cycle effect was found (*F*_23,184_ = 4.880, *p* < 0.0001), but no significant genotype or interaction effects were observed (respectively, *F*_1,8_ = 0.7295, *p* = 0.418; *F*_23,184_ = 0.5618, *p* = 0.948). Data are means ± SEM of 5 mice in each condition for each hour across the 24 h distribution. Significance is based on 2-way ANOVAs using multiple comparisons. Gray backgrounds indicate the 12 h dark period.

To determine if the findings for male CaMKIV-OE aged mice held true for their female counterparts, we next quantified the total amount of time spent in each vigilance state in aged female mice overexpressing CaMKIV compared to age-matched female wild-type mice. During the full 24 h recording, aged CaMKIV-OE female mice displayed no significant differences in the amount of time spent in each of the three vigilance states compared to their aged wild-type female counterparts (Figure 4A). To explore potential time-of-day effects masked in the broader 12 h intervals, additional analysis was performed in 6 h bins; however, no significant differences in the amount of time spent in each of the three vigilance states were found (data not shown). Furthermore, when analyzing the 24 h distribution of sleep–wake states, aged CaMKIV-OE female mice demonstrated no significant differences for each individual hour between genotypes for all three vigilance states compared to aged wild-type female mice (Figure 4B). Considered alongside the results obtained for their aged male counterparts, this data indicates that the effects of CaMKIV overexpression are differential based on sex.

### 3.5 CaMKIV overexpression promotes sleep fragmentation in young adult male mice

**Figure 5:**
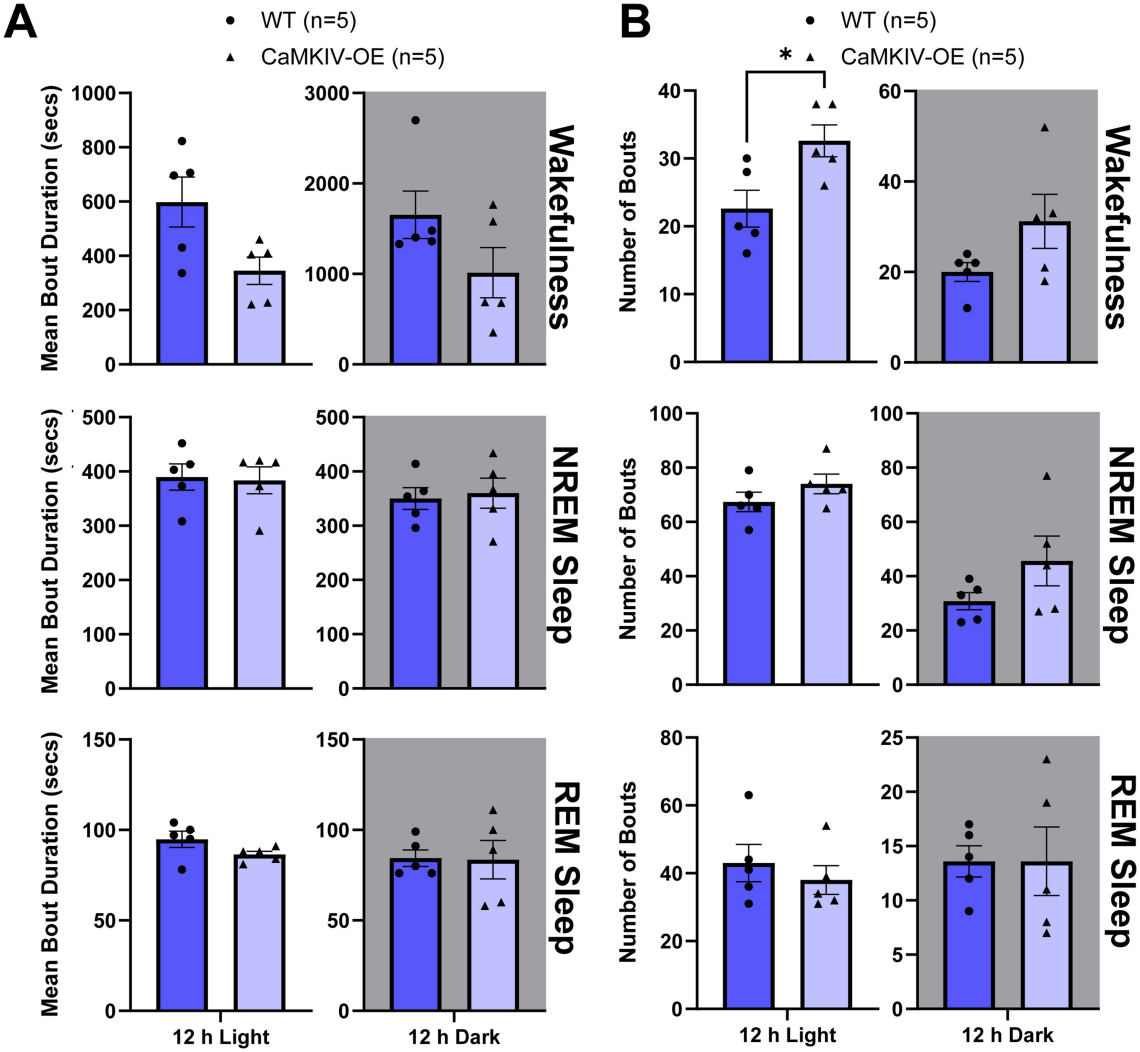
Young adult CaMKIV-OE male mice had an increased number of bouts of wakefulness during the 12 h light period. Parameters of vigilance state consolidation/fragmentation of young adult WT and CaMKIV-OE male mice quantified for the 12 h light and 12 h dark periods using EEG/EMG recordings. **(A)** Mean duration of individual bouts of vigilance states (wake, NREM sleep, and REM sleep). No significant genotype effects were observed for 12 h light wake (*p* = 0.0952), 12 h dark wake (*p* = 0.421), 12 h light NREM sleep (*p* = 0.857), 12 h dark NREM sleep (*p* = 0.690), 12 h light REM sleep (*p* = 0.135), and 12 h dark REM sleep (*p* = 0.952). **(B)** Number of individual bouts of vigilance states (wake, NREM sleep, and REM sleep). A significant genotype effect was found for 12 h light wake (*p = 0.0397). No significant genotype effects were observed for 12 h dark wake (*p* = 0.286), 12 h light NREM sleep (*p* = 0.238), 12 h dark NREM sleep (*p* = 0.222), 12 h light REM sleep (*p* = 0.490), and 12 h dark REM sleep (*p* > 0.841). Data are means ± SEM of 5 mice in each condition. Asterisks indicate significant comparisons between genotypes (*p < 0.05; Mann–Whitney U test). Gray backgrounds indicate the 12 h dark period.

Next, we examined sleep quality in young adult male mice by quantifying the total number of bouts and bout duration for each vigilance state. Although there were no significant differences in the mean bout duration of each vigilance state between genotypes (Figure 5A), the number of individual bouts of wakefulness were increased in young adult CaMKIV-OE male mice compared to young adult wild-type male mice during the 12 h light period (Figure 5B). This increased number of bouts of wakefulness during the inactive phase of the mice is an indicator of more frequent transitions between sleep–wake states, suggesting that sleep is more fragmented in young adult mice overexpressing CaMKIV.

### 3.6 CaMKIV overexpression does not alter sleep–wake architecture in young adult female mice

**Figure 6:**
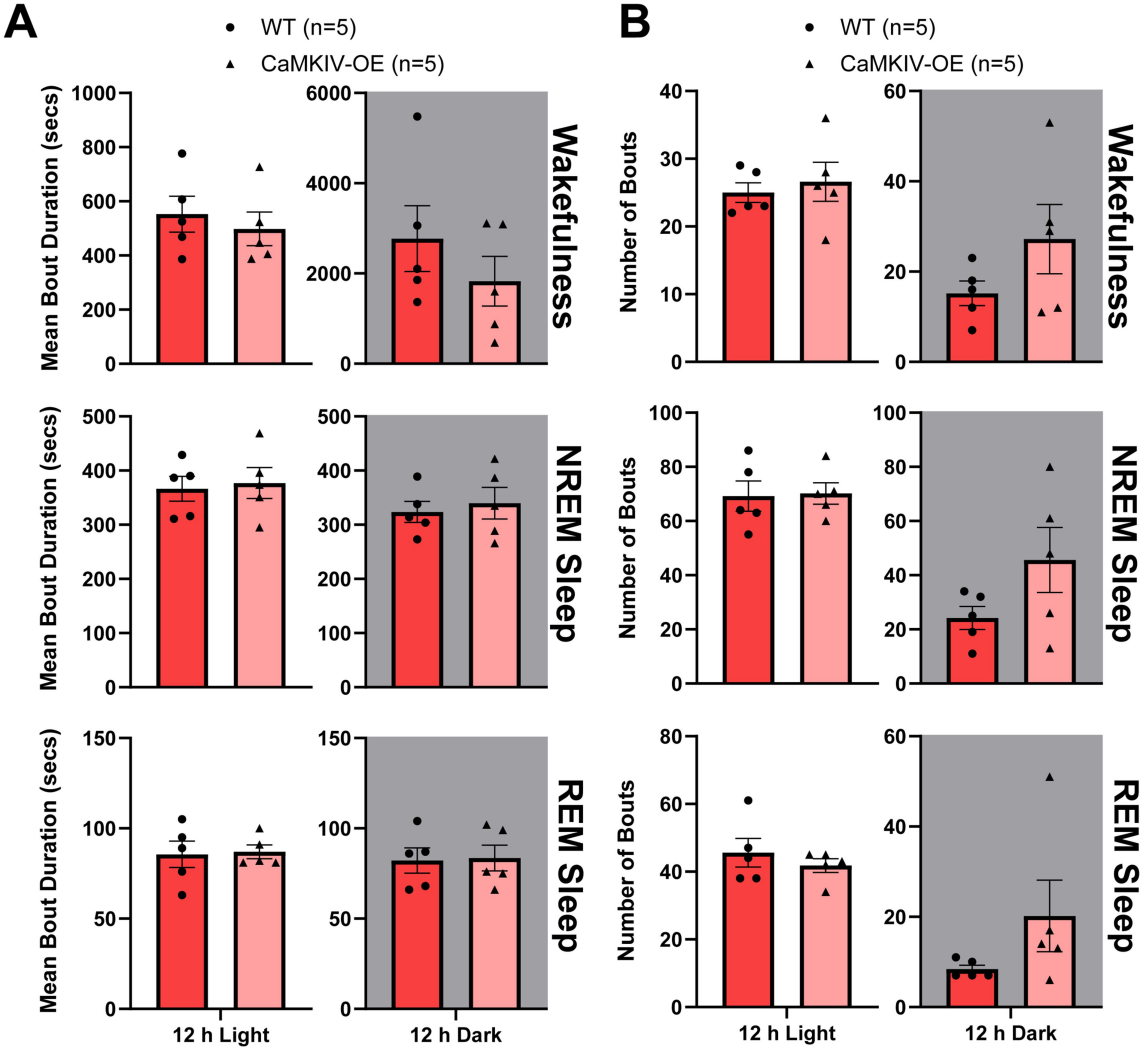
Young adult CaMKIV-OE female mice had no significant differences in vigilance state bout duration or bout number for the 12 h light and the 12 h dark periods. Parameters of vigilance state consolidation/fragmentation of young adult WT and CaMKIV-OE female mice quantified for the 12 h light and 12 h dark periods using EEG/EMG recordings. **(A)** Mean duration of individual bouts of vigilance states (wake, NREM sleep, and REM sleep). No significant genotype effects were observed for 12 h light wake (*p* = 0.548), 12 h dark wake (*p* = 0.548), 12 h light NREM sleep (*p* > 0.999), 12 h dark NREM sleep (*p* > 0.999), 12 h light REM sleep (*p* = 0.952), and 12 h dark REM sleep (*p* > 0.999). **(B)** Number of individual bouts of vigilance states (wake, NREM sleep, and REM sleep). No significant genotype effects were observed for 12 h light wake (*p* = 0.762), 12 h dark wake (*p* = 0.333), 12 h light NREM sleep (*p* = 0.841), 12 h dark NREM sleep (*p* = 0.222), 12 h light REM sleep (*p* = 0.690), and 12 h dark REM sleep (*p* = 0.151). Data are means ± SEM of 5 mice in each condition. Gray backgrounds indicate the 12 h dark period.

To assess the sleep quality of young adult CaMKIV-OE female mice, the total number of bouts and bout duration for each vigilance state in CaMKIV-OE mice were compared to wild-type, revealing no significant differences in either measure (Figure 6). Thus, the overexpression of CaMKIV was not shown to cause sleep fragmentation or consolidation in young adult female mice. This finding was consistent with the observation that the overexpression of CaMKIV in young adult female mice does not alter the percentage of time spent in each vigilance state, suggesting overall that overexpression of CaMKIV in young adult female mice has no significant effect on sleep–wake architecture. In conjunction with the young adult male mice results, this data further supports the conclusion that CaMKIV overexpression has sexually dimorphic effects on sleep–wake regulation.

### 3.7 CaMKIV overexpression promotes wake fragmentation in aged male mice

**Figure 7:**
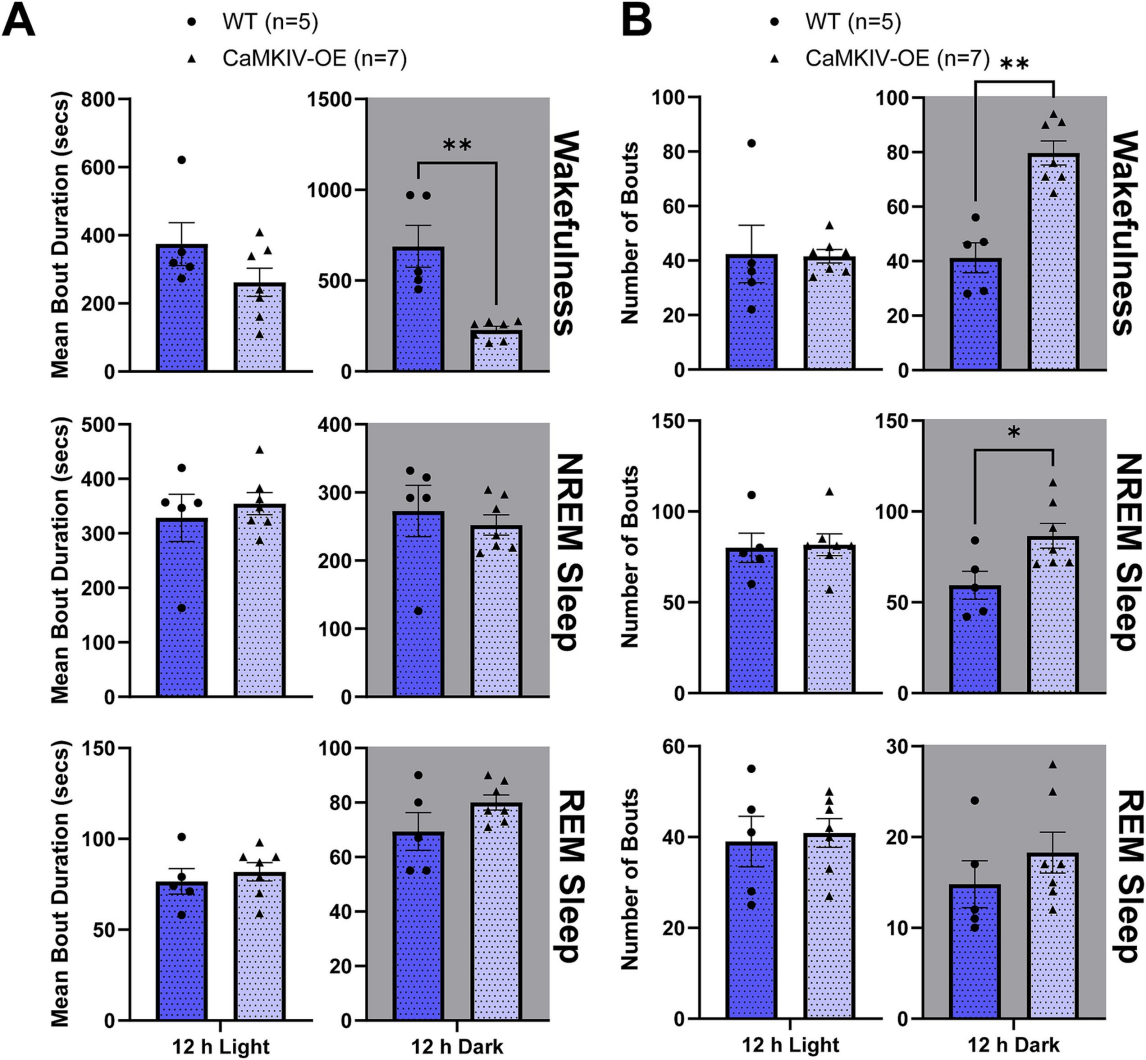
Aged CaMKIV-OE male mice had decreased wake bout duration and an increased number of bouts of wakefulness and NREM sleep during the 12 h dark period. Parameters of vigilance state consolidation/fragmentation of aged WT and CaMKIV-OE male mice quantified for the 12 h light and 12 h dark periods using EEG/EMG recordings. **(A)** Mean duration of individual bouts of vigilance states (wake, NREM sleep, and REM sleep). A significant genotype effect was found for 12 h dark wake (**p = 0.00253). No significant genotype effects were observed for 12 h light wake (*p* = 0.343), 12 h light NREM sleep (*p* > 0.999), 12 h dark NREM sleep (*p* = 0.322), 12 h light REM sleep (*p* = 0.556), 12 h dark REM sleep (*p* = 0.278). **(B)** Number of individual bouts of vigilance states (wake, NREM sleep, and REM sleep). Significant genotype effects were found for 12 h dark wake (**p = 0.00126) and 12 h dark NREM sleep (*p = 0.0278). No significant genotype effects were observed for 12 h light wake (*p* = 0.364), 12 h light NREM sleep (*p* = 0.428), 12 h light REM sleep (*p* = 0.789), and 12 h dark REM sleep (*p* = 0.215). Data are means ± SEM of 5 or 7 mice in each condition. Asterisks indicate significant comparisons between genotypes (**p < 0.01, *p < 0.05; Mann–Whitney *U* test). Gray backgrounds indicate the 12 h dark period.

The total number of bouts and bout duration were then quantified for each vigilance state for aged male mice. In aged CaMKIV-OE male mice, the mean bout duration of each bout of wakefulness was decreased compared to aged wild-type male mice during the 12 h dark period (Figure 7A). Furthermore, the number of individual bouts of wakefulness and NREM sleep were increased compared to aged wild-type male mice during the 12 h dark period (Figure 7B). In contrast to the sleep fragmentation observed during the light period in their young adult male counterparts, this larger number of shorter bouts of wakefulness observed during the dark period of the aged male mice reveals wake that is more fragmented in aged male mice overexpressing CaMKIV.

### 3.8 CaMKIV overexpression promotes NREM sleep consolidation in aged female mice

**Figure 8:**
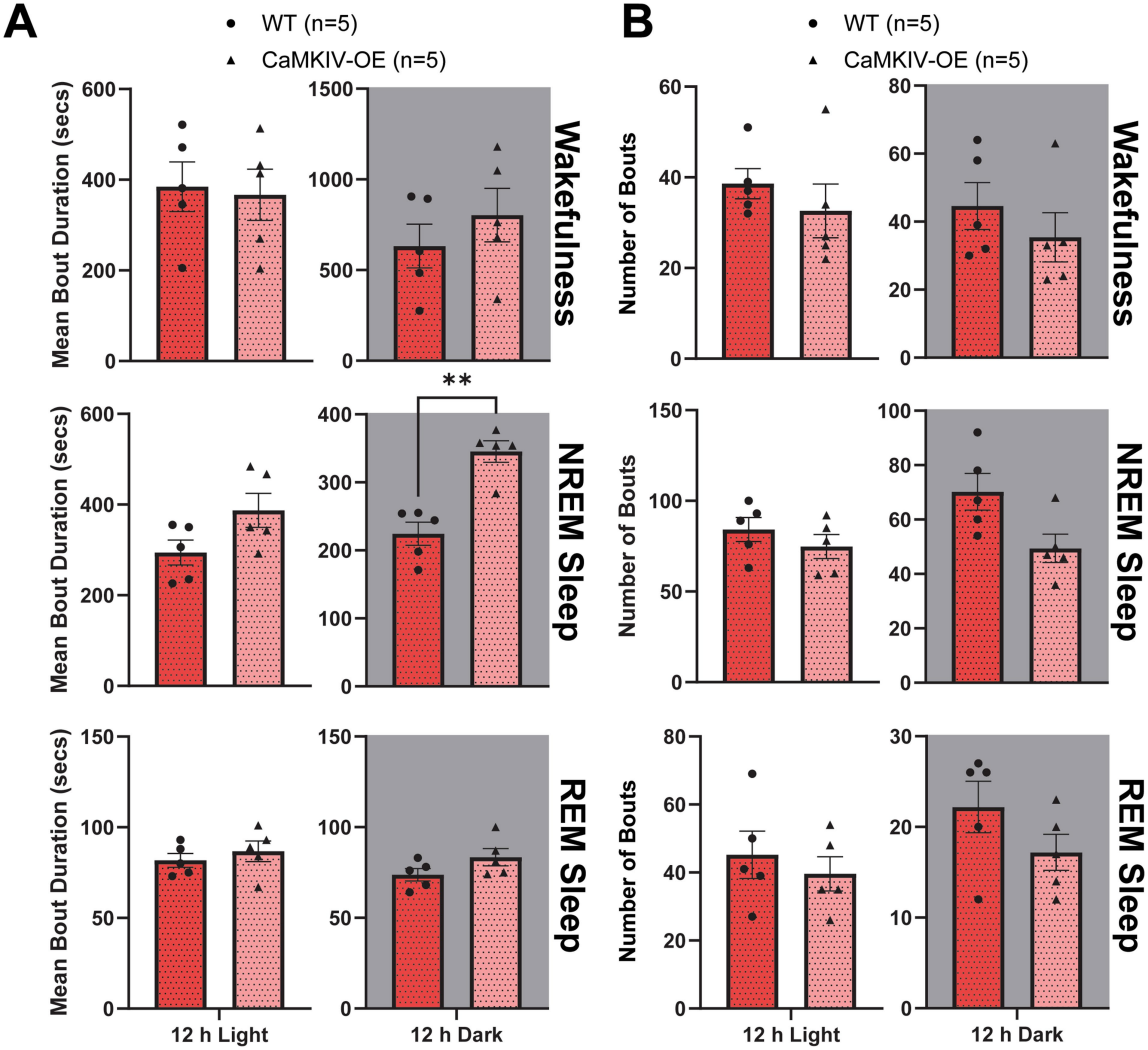
Aged CaMKIV-OE female mice had increased NREM sleep bout duration during the 12 h dark period. Parameters of vigilance state consolidation/fragmentation of aged WT and CaMKIV-OE female mice quantified for the 12 h light and 12 h dark periods using EEG/EMG recordings. **(A)** Mean duration of individual bouts of vigilance states (wake, NREM sleep, and REM sleep). A significant genotype effect was found for 12 h dark NREM sleep (**p = 0.00794). No significant genotype effects were observed for 12 h light wake (*p* = 0.841), 12 h dark wake (*p* = 0.421), 12 h light NREM sleep (*p* = 0.238), 12 h light REM sleep (*p* = 0.452), and 12 h dark REM sleep (*p* = 0.310). **(B)** Number of individual bouts of vigilance states (wake, NREM sleep, and REM sleep). No significant genotype effects were observed for 12 h light wake (*p* = 0.254), 12 h dark wake (*p* = 0.421), 12 h light NREM sleep (*p* = 0.310), 12 h dark NREM sleep (*p* = 0.0556), 12 h light REM sleep (*p* = 0.516), 12 h dark REM sleep (*p* = 0.214). Data are means ± SEM of 5 mice in each condition. Asterisks indicate significant comparisons between genotypes (**p < 0.01, *p < 0.05; Mann–Whitney *U* test). Gray backgrounds indicate the 12 h dark period.

Lastly, to assess the sleep quality of aged CaMKIV-OE female mice, the total number of bouts and bout duration were quantified for each vigilance state. In aged CaMKIV-OE female mice, the mean bout duration of each individual bout of NREM sleep was increased compared to aged wild-type female mice during the 12 h dark period (Figure 8A). These significantly longer episodes of NREM sleep during the inactive phase of the mice suggest that aged female mice experience more consolidated sleep following the overexpression of CaMKIV. However, there were no significant differences in the total number of individual bouts of each vigilance state in aged CaMKIV-OE female mice compared to young adult wild-type female mice (Figure 8B). This distinct sleep phenotype compared to their male counterparts is consistent with what we demonstrated in our previous data: the effect of CaMKIV overexpression is differential based on sex.

## 4 Discussion

Analysis of the sleep–wake architecture of young adult and aged mice overexpressing CaMKIV in the forebrain revealed that this CREB-activating protein has the ability to alter sleep patterns, particularly during the 12 h dark period. While overexpression of CaMKIV produced significant differences in the percentage of time aged male mice spent in each vigilance state, it did not produce a significant difference in their young adult male counterparts. Further, overexpression of CaMKIV had no significant impact on the percentage of time female mice spent in each vigilance state, even after the aging process. Thus, here we report that overexpression of CaMKIV can differentially influence sleep–wake architecture depending on sex and age. This finding ultimately builds upon previous literature indicating that CaMKIV enhances the quality of sleep, and suggests a potential means to amplify sleep duration in aged mice.

Across both sleep analysis measures—percentage of time spent in each vigilance state, as well as vigilance state bout quantity and duration—CaMKIV most frequently affected NREM sleep, as opposed to REM sleep. Slow-wave NREM sleep has been shown to play a crucial role in memory consolidation (Walker, 2009; Rasch and Born, 2013). Considered alongside the fact that CaMKIV overexpression has been shown to improve memory in young adult male and female mice and restore memory deficits in aged mice (Fukushima et al., 2008; Wu et al., 2008), it is possible that a mechanism by which CaMKIV is able to enhance memory is through the promotion of memory consolidation-facilitating NREM sleep. Indeed, CaMKIV overexpression in the forebrain of mice has previously been shown to enhance cortical delta oscillations—a type of brain wave pattern that has been implicated in promoting memory consolidation—during post-learning NREM sleep, as well as amplify 4–7.5 Hz oscillations during trace fear learning (Steenland et al., 2010). Our study, which has shown that CaMKIV can enhance sleep in aged male mice, primarily through an increase in NREM sleep, further suggests an intimate link between sleep–wake architecture and memory consolidation.

In addition to an increase in total NREM sleep duration, aged male mice that overexpress CaMKIV also experienced more bouts of wakefulness and spent less time awake per individual bout during the 12 h dark period. Mice are nocturnal animals that experience sleep that is much more fragmented than humans, and also display polyphasic sleep, meaning that they tend to sleep in multiple short, successive bouts throughout the 24 h light cycle (Hiyoshi et al., 2014). Thus, it is unsurprising that the increase in the total sleep duration of CaMKIV-OE aged male mice was coupled with an increased observance of sleep–wake fragmentation—the natural sleep–wake pattern of mice. However, it is notable that while the increase in NREM sleep, REM sleep, and wake fragmentation observed over the 24 h recording period in the aged male mice tended to be localized to the 12 h dark period of the light cycle, or the active phase of the mice, the sleep fragmentation observed in young adult male mice was localized to the 12 h light period, or the inactive phase of the mice. Interestingly, most of the natural effects of aging on the sleep–wake cycle of mice—including an increase in NREM sleep and sleep–wake fragmentation—also occur during the dark period (Wimmer et al., 2013; Soltani et al., 2019), suggesting that CaMKIV overexpression amplifies many of the sleep–wake changes associated with normal mouse aging. Regardless of these age-related distinctions, it is notable that in this study, the expression of a protein most well-known for its role in promoting memory consolidation, CaMKIV, was able to alter sleep–wake architecture. Inherent in this finding is the idea that hyperactive learning can alter the regulation of sleep, further suggesting a bidirectional relationship between sleep and memory, beyond the more well-documented impact of altered sleep patterns on memory consolidation.

Another interesting observation of this study was that, unlike their male counterparts, aged female mice overexpressing CaMKIV experienced more consolidated NREM sleep, as reflected by the increase in NREM bout duration during the 12 h dark period of the light cycle. Overall, this finding was consistent with the idea that CaMKIV overexpression appears to enhance the quality of sleep in aged mice, albeit differentially between males and females. When coupled with the finding that young adult and aged female mice overexpressing CaMKIV display no significant differences in total time spent in each vigilance state, it seems as though the sleep—and potentially memory—enhancing effects of CaMKIV overexpression arise via different mechanisms in male and female mice. Sexual dimorphism has already been observed in the memory-enhancing mechanisms of other members of the CaMK cascade. For example, CaMKKα, which phosphorylates and activates both CaMKI and CaMKIV in response to calcium influx, has been shown to be essential for contextual fear memory formation in male, but not female, mice due to male-specific, learning-induced upregulation of brain-derived neurotrophic factor transcripts (Mizuno et al., 2006). Further, a majority of studies examining the role of CaMKIV in both memory and the regulation of sleep, such as the previously discussed ability of CaMKIV overexpression to enhance delta oscillations during NREM sleep, were conducted in male mice, and therefore may not be entirely applicable to female mice. Thus, future studies aimed at investigating the mechanisms that enhance sleep quality and memory in CaMKIV-OE mice should incorporate both male and female mice to elucidate the mechanistic basis of these differential effects.

Further, while our study supports a role for CaMKIV in positively regulating sleep–wake architecture, it is important to note that we are unable to fully determine whether these changes are promoted only by the preventative accumulation of CaMKIV over the course of the lifespan of CaMKIV-OE mice, or if acute expression of CaMKIV in aged mice already displaying age-associated sleep disturbances could also reverse age-associated changes in sleep–wake architecture. Previous research on CaMKIV, including this study, has yet to investigate CaMKIV overexpression beyond transgenic models, making the effect of transient activation or overexpression of CaMKIV on sleep–wake architecture an interesting target for future studies. It is also notable that while previous studies have suggested a role for CREB in promoting arousal (Hendricks et al., 2001; Graves et al., 2003; Wimmer et al., 2021), our study, involving overexpression of CaMKIV, has implicated this CREB-activating protein in promoting NREM sleep in male mice. Thus, future research on the CaMK/CREB pathway should also aim to elucidate the mechanistic basis of this difference in the regulation of sleep–wake states.

In summary, the memory-enhancing protein CaMKIV appears to also positively regulate sleep–wake architecture in aged male mice, raising the possibility that CaMKIV improves memory consolidation by enhancing NREM sleep. In this study, we demonstrate that although CaMKIV can enhance sleep, sleep is differentially affected in both male and female mice, potentially due to differences in physiology, such as natural hormonal fluctuations experienced by female mice. Given the fact that CaMKIV has been implicated in memory consolidation and now the regulation of sleep–wake architecture, our study reiterates the importance of continually studying the mechanisms that underlie age-related changes in the regulation of sleep, which could reveal more therapeutic targets that reverse age-related changes in cognitive function. Lastly, our findings further encourage future research into examining the sexually dimorphic effects of CaMKIV on sleep–wake architecture by studying the cellular and molecular mechanisms underlying the differential sleep physiology between male and female mice.

## Data Availability

The original contributions presented in the study are included in the article/Supplementary material, further inquiries can be directed to the corresponding author.

## References

[ref1] Chrivia J. C. Kwok R. P. S. Lamb N. Hagiwara M. Montminy M. R. Goodman R. H. (1993). Phosphorylated CREB binds specifically to the nuclear protein CBP. Nature 365, 855–859. doi: 10.1038/365855a0, PMID: 8413673

[ref2] Cirelli C. Gutierrez C. M. Tononi G. (2004). Extensive and divergent effects of sleep and wakefulness on brain gene expression. Neuron 41, 35–43. doi: 10.1016/S0896-6273(03)00814-6, PMID: 14715133

[ref3] Cirelli C. Tononi G. (2000). Differential expression of plasticity-related genes in waking and sleep and their regulation by the noradrenergic system. J. Neurosci. Off. J. Soc. Neurosci. 20, 9187–9194. doi: 10.1523/JNEUROSCI.20-24-09187.2000, PMID: 11124996 PMC6773024

[ref4] Fukushima H. Maeda R. Suzuki R. Suzuki A. Nomoto M. Toyoda H.. (2008). Upregulation of calcium/calmodulin-dependent protein kinase IV improves memory formation and rescues memory loss with aging. J. Neurosci. Off. J. Soc. Neurosci. 28, 9910–9919. doi: 10.1523/JNEUROSCI.2625-08.2008, PMID: 18829949 PMC6671266

[ref5] Graves L. A. Heller E. A. Pack A. I. Abel T. (2003). Sleep deprivation selectively impairs memory consolidation for contextual fear conditioning. Learn. Memory 10, 168–176. doi: 10.1101/lm.48803, PMID: 12773581 PMC202307

[ref6] Graves L. A. Hellman K. Veasey S. Blendy J. A. Pack A. I. Abel T. (2003). Genetic evidence for a role of CREB in sustained cortical arousal. J. Neurophysiol. 90, 1152–1159. doi: 10.1152/jn.00882.2002, PMID: 12711709

[ref7] Hendricks J. C. Williams J. A. Panckeri K. Kirk D. Tello M. Yin J. C. P.. (2001). A non-circadian role for cAMP signaling and CREB activity in Drosophila rest homeostasis. Nat. Neurosci. 4, 1108–1115. doi: 10.1038/nn743, PMID: 11687816

[ref8] Hiyoshi H. Terao A. Okamatsu-Ogura Y. Kimura K. (2014). Characteristics of sleep and wakefulness in wild-derived inbred mice. Exp. Anim. 63, 205–213. doi: 10.1538/expanim.63.205, PMID: 24770646 PMC4160977

[ref9] Loerch P. M. Lu T. Dakin K. A. Vann J. M. Isaacs A. Geula C.. (2008). Evolution of the aging brain transcriptome and synaptic regulation. PLoS One 3:e 3329. doi: 10.1371/journal.pone.0003329, PMID: 18830410 PMC2553198

[ref10] Lonze B. E. Ginty D. D. (2002). Function and regulation of CREB family transcription factors in the nervous system. Neuron 35, 605–623. doi: 10.1016/s0896-6273(02)00828-0, PMID: 12194863

[ref11] Lu T. Pan Y. Kao S. Y. Li C. Kohane I. Chan J.. (2004). Gene regulation and DNA damage in the ageing human brain. Nature 429, 883–891. doi: 10.1038/nature02661 15190254

[ref12] Mander B. A. Winer J. R. Walker M. P. (2017). Sleep and Human Aging. Neuron 94, 19–36. doi: 10.1016/j.neuron.2017.02.004, PMID: 28384471 PMC5810920

[ref13] Matthews R. P. Guthrie C. R. Wailes L. M. Zhao X. Means A. R. McKnight G. (1994). Calcium/calmodulin-dependent protein kinase types II and IV differentially regulate CREB-dependent gene expression. Mol. Cell. Biol. 14, 6107–6116. doi: 10.1128/mcb.14.9.6107-6116.1994, PMID: 8065343 PMC359137

[ref14] Mayr B. Montminy M. (2001). Transcriptional regulation by the phosphorylation-dependent factor CREB. Nat. Rev. Mol. Cell Biol. 2, 599–609. doi: 10.1038/35085068 11483993

[ref15] McKillop L. E. Vyazovskiy V. V. (2020). Sleep and ageing: from human studies to rodent models. Curr. Opin. Physio. 15, 210–216. doi: 10.1016/j.cophys.2020.03.004, PMID: 32467862 PMC7255885

[ref16] Mizuno K. Ris L. Sánchez-Capelo A. Godaux E. Giese K. P. (2006). Ca2+/calmodulin kinase kinase α is dispensable for brain development but is required for distinct memories in male, though not in female, mice. Mol. Cell. Biol. 26, 9094–9104. doi: 10.1128/MCB.01221-06, PMID: 17015468 PMC1636825

[ref17] Rasch B. Born J. (2013). About sleep’s role in memory. Physiol. Rev. 93, 681–766. doi: 10.1152/physrev.00032.2012, PMID: 23589831 PMC3768102

[ref18] Soltani S. Chauvette S. Bukhtiyarova O. Lina J. M. Dubé J. Seigneur J.. (2019). Sleep-wake cycle in young and older mice. Front. Syst. Neurosci. 13:51. doi: 10.3389/fnsys.2019.00051, PMID: 31611779 PMC6769075

[ref19] Steenland H. W. Wu V. Fukushima H. Kida S. Zhuo M. (2010). CaMKIV over-expression boosts cortical 4-7 Hz oscillations during learning and 1-4 Hz delta oscillations during sleep. Mol. Brain 3:16. doi: 10.1186/1756-6606-3-16, PMID: 20497541 PMC2888801

[ref20] Toescu E. C. Vreugdenhil M. (2010). Calcium and normal brain ageing. Cell Calcium 47, 158–164. doi: 10.1016/j.ceca.2009.11.013, PMID: 20045187

[ref21] US Department of Health and Human Services A Profile of Older Americans: 2012 (n.d.). Available at: https://acl.gov/sites/default/files/aging%20and%20disability%20in%20america/2012profile.pdf

[ref22] US Department of Health and Human Services A Profile of Older Americans: 2023 (2024). Available at: https://acl.gov/sites/default/files/Profile%20of%20OA/ACL_ProfileOlderAmericans2023_508.pdf

[ref23] Walker M. P. (2009). The role of slow wave sleep in memory processing. J. Clin. Sleep Med. 5, S20–S26. doi: 10.5664/jcsm.5.2S.S20 19998871 PMC2824214

[ref24] Wimmer M. E. Cui R. Blackwell J. M. Abel T. (2021). Cyclic AMP response element-binding protein is required in excitatory neurons in the forebrain to sustain wakefulness. Sleep 44:zsaa267. doi: 10.1093/sleep/zsaa267, PMID: 33277644 PMC8193557

[ref25] Wimmer M. E. Rising J. Galante R. J. Wyner A. Pack A. I. Abel T. (2013). Aging in mice reduces the ability to sustain sleep/wake states. PLoS One 8:e81880. doi: 10.1371/journal.pone.0081880, PMID: 24358130 PMC3864844

[ref26] Wu L.-J. Zhang X. H. Fukushima H. Zhang F. Wang H. Toyoda H.. (2008). Genetic enhancement of trace fear memory and cingulate potentiation in mice overexpressing Ca2+/calmodulin-dependent protein kinase IV. Eur. J. Neurosci. 27, 1923–1932. doi: 10.1111/j.1460-9568.2008.06183.x, PMID: 18412613

[ref27] Yu X.-W. Oh M. M. Disterhoft J. F. (2017). CREB, cellular excitability, and cognition: implications for aging. Behav. Brain Res. 322, 206–211. doi: 10.1016/j.bbr.2016.07.042, PMID: 27478142 PMC5274584

[ref28] Zagaar M. A. Dao A. T. Alhaider I. A. Alkadhi K. A. (2016). Prevention by regular exercise of acute sleep deprivation-induced impairment of late phase LTP and related signaling molecules in the dentate gyrus. Mol. Neurobiol. 53, 2900–2910. doi: 10.1007/s12035-015-9176-4, PMID: 25902862

[ref29] Zagaar M. Dao A. Levine A. Alhaider I. Alkadhi K. (2013). Regular exercise prevents sleep deprivation associated impairment of long-term memory and synaptic plasticity in the CA1 area of the hippocampus. Sleep 36, 751–761. doi: 10.5665/sleep.2642, PMID: 23633758 PMC3624830

