# Supplementary material for: Sleep is enhanced in aged male mice that overexpress calcium/calmodulin-dependent protein kinase IV
Source: Front Neurosci. 2025 Jun 3;19:1596602. doi: 10.3389/fnins.2025.1596602 (PMC12170523; doi:10.3389/fnins.2025.1596602)
Supplement: Supplementary file 1 [file Data_Sheet_1.docx]

**
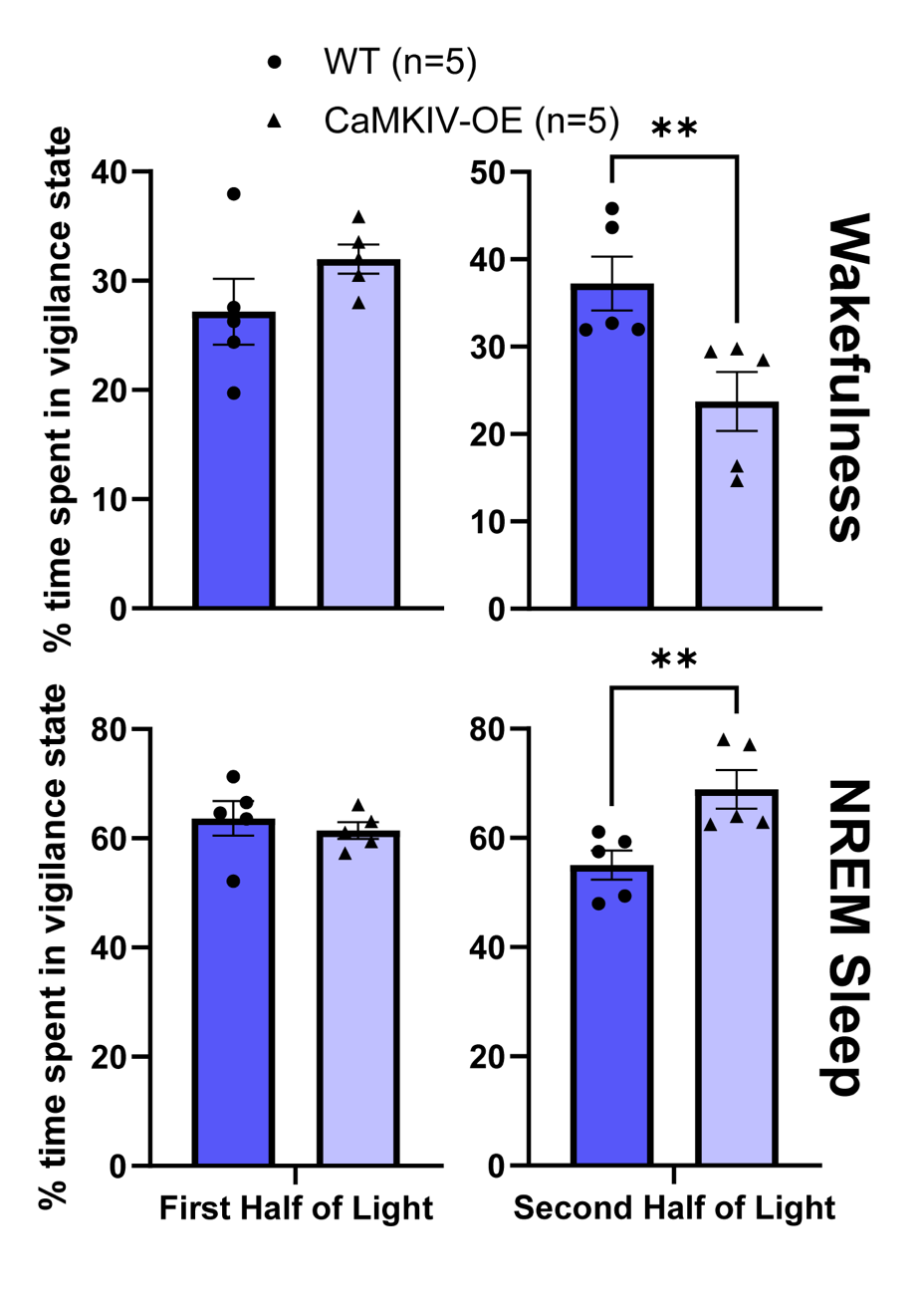
**

**Supplementary Figure 1. Young adult CaMKIV-OE male mice had decreased wakefulness and increased NREM sleep during the second half of the light cycle****.** The percentage of time young adult male mice spent in wake and NREM sleep for the first (ZT 0-6) and second (ZT 6-12) half of the light cycle is shown. A significant genotype effect was found for wake (***p* = 0.00794) and NREM sleep (***p* = 0.00794) during the second half of the light cycle. No significant genotype effect was observed for wake (*p* = 0.151) and NREM sleep (*p* = 0.310) during the first half of the light cycle. Data are means ± SEM of 5 mice in each condition. Asterisks indicate significant comparisons between genotypes (**: *p* < 0.01; *: *p* < 0.05, Mann-Whitney U Test). The percentage of time young adult male mice spent in REM sleep during the light cycle, as well as the percentage of time young adult male mice spent in wake, NREM sleep, and REM sleep during the dark cycle, were also analyzed in 6-hour bins, but no significant differences were observed (data not shown).

**
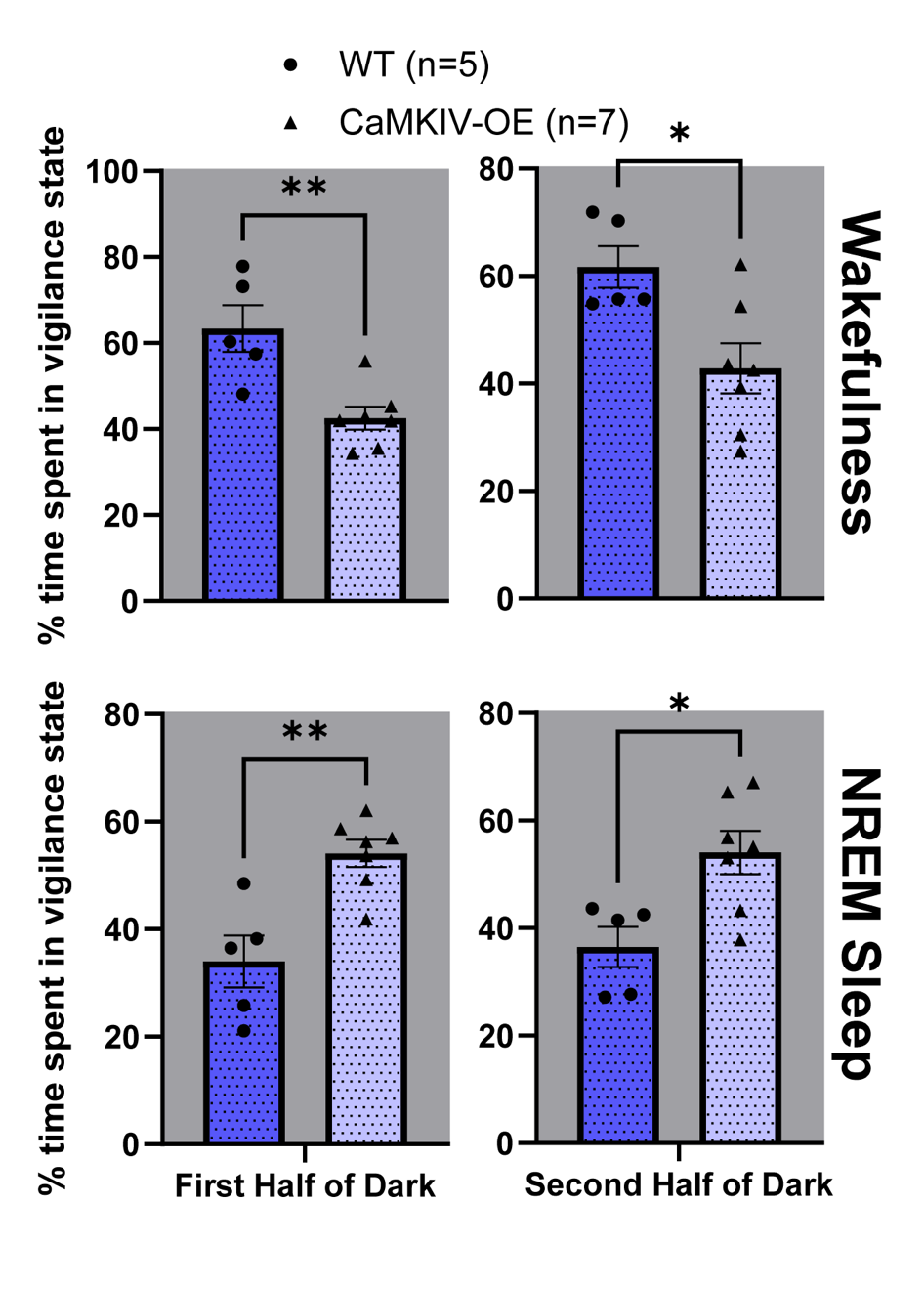
**

**Supplementary Figure 2. Aged CaMKIV-OE male mice had decreased wakefulness during the first and second half of the dark cycle, and increased NREM sleep during the first and second half of the dark cycle.** Percentage of time aged male mice spent in wake and NREM sleep for the first (ZT 12-18) and second (ZT 18-24) half of the dark cycle is shown. A significant genotype effect was found for wake (***p* = 0.00505) and NREM sleep (***p* = 0.00505) during the first half of the dark period, as well as for wake (**p* = 0.0152) and NREM sleep (**p* = 0.0303) during the second half of the dark period. Data are means ± SEM of 5 or 7 mice in each condition. Asterisks indicate significant comparisons between genotypes (**: *p* < 0.01; *: *p* < 0.05, Mann-Whitney U Test). The percentage of time aged male mice spent in wake, NREM sleep, and REM sleep during the light cycle, as well as the percentage of time aged male mice spent in REM sleep during the dark cycle, were also analyzed in 6-hour bins, but no significant differences were observed (data not shown).
